# Management of Malnutrition Based on Multidisciplinary Team Decision-Making in Chinese Older Adults (3M Study): A Prospective, Multicenter, Randomized, Controlled Study Protocol

**DOI:** 10.3389/fnut.2022.851590

**Published:** 2022-05-09

**Authors:** Tong Ji, Li Zhang, Rui Han, Linlin Peng, Shanshan Shen, Xiaolei Liu, Yanqing Shi, Xujiao Chen, Qiong Chen, Yun Li, Lina Ma

**Affiliations:** ^1^Department of Geriatrics, Xuanwu Hospital Capital Medical University, Beijing, China; ^2^Department of Geriatrics, Xiangya Hospital Central South University, Changsha, China; ^3^National Clinical Research Center for Geriatric Disorders, Xiangya Hospital, Central South University, Changsha, China; ^4^Department of Geriatrics, Zhejiang Hospital, Hangzhou, China; ^5^Department of Geriatrics, West China Hospital Sichuan University, Chengdu, China; ^6^Department of Geriatrics, Fujian Medical University Union Hospital, Fuzhou, China

**Keywords:** malnutrition, older adults, multidisciplinary team, randomized control study, protocol

## Abstract

**Background:**

In hospital settings, malnutrition affects 30–50% of aged inpatients and is related to a higher risk of hospital complications and death. This study aims to demonstrate the effectiveness of a tailored optimum nutritional therapy in malnourished, elderly inpatients based on multidisciplinary team recommendations in hopes of decreasing the incidence of deleterious clinical outcomes.

**Methods and Design:**

This trial will be a multicenter, open-label, randomized control trial conducted in the geriatric wards of at least five hospitals in five different regions. We aim to include 500 inpatients over the age of 60 with or at risk of malnutrition based on a Mini Nutritional Assessment Short-Form (MNA-SF) score of ≤ 11 points and the Global Leadership Initiative on Malnutrition with an expected length of stay of ≥ 7 days. Eligible inpatients will be randomized into a 1:1 ratio, with one receiving a multidisciplinary team intervention and the other receiving standard medical treatment or care alone. A structured comprehensive assessment of anthropometry, nutritional status, cognition, mood, functional performance, and quality of life will be conducted twice. These assessments will take place on the day of group allocation and 1 year after discharge, and a structured screening assessment for elderly malnutrition will be conducted at 3 and 6 months after discharge using the MNA-SF. The primary outcome will be nutritional status based on changes in MNA-SF scores at 3, 6 months, and 1 year. The secondary outcome will be changes in cognition, mood, functional status, length of hospital stay, and all-cause mortality 1 year after discharge.

**Discussion:**

Guided by the concept of interdisciplinary cooperation, this study will establish a multidisciplinary nutrition support team that will develop an innovative intervention strategy that integrates nutritional screenings, evaluations, education, consultation, support, and monitoring. Moreover, nutritional intervention and dietary fortification will be provided to hospitalized elderly patients with or at risk of malnutrition. The nutrition support team will formulate a clinical map for malnutrition in elderly patients with standardized diagnosis and treatment for malnutrition in this population.

**Clinical Trial Registration:**

[www.ClinicalTrials.gov], identifier [ChiCTR2200055331].

## Introduction

Malnutrition is caused by nutrient deficiency, with or without inflammation, and results in increased metabolic demand and changes in body composition and cell mass, especially the reduction of fat-free weight (1). These changes negatively affect physical and psychological functions as well as clinical outcomes (2). Human organ structures degenerate naturally during the process of aging, and the functional reserve of the most organs of old age decreases, mainly due to decreased digestive capacity, poor dental status, taste alteration, and changes in human composition (reduction of lean tissue) (3, 4). This process results in insufficient food intake and absorption, metabolic disorders, and increased likelihood of malnutrition in aged populations (5). In addition, various comorbidities and polypharmacy play a role in malnutrition in the elderly. Malnutrition is associated with adverse clinical outcomes, such as a decline in physical and cognitive function, depression, frailty, sarcopenia, longer hospital stays, impaired quality of life, increased risk of infection, and mortality, which may result in heavy burdens on the individuals, their families, and society (6–8). Previous studies have revealed that malnutrition affects as many as 30–50% of elderly patients in nursing homes and acute care settings, highlighting the importance of assessing malnutrition in this population (9–11).

In the past, due to the lack of unified diagnostic criteria, it was difficult to standardize the diagnosis and treatment of malnutrition. In this context, the Global Leadership Initiative on Malnutrition (GLIM) developed the first international consensus, which was of great significance for standardizing the diagnosis, treatment, and the use of medical insurance for malnutrition (12). The GLIM criteria proposed a two-step approach: screening to identify the risk of malnutrition first and then assessing for a diagnosis and grading the severity of malnutrition. According to the GLIM criteria, the Mini Nutritional Assessment Short-Form (MNA-SF) is recommended for malnutrition risk screenings in older populations (12). The malnutrition diagnosis standard includes a combination of at least one of the phenotypic criteria (weight loss, reduced body mass index [BMI], or reduced muscle mass) and one of the etiologic criteria (reduced food intake/assimilation or disease burden/inflammation) recommended by the GLIM. Several clinical studies were conducted in both China and abroad as soon as the GLIM criteria were published. A prospective cohort study showed that the GLIM criteria for malnutrition diagnosis in inpatients was fully validated and could be applied in clinical practice (13). At present, there is a lack of interventional research to demonstrate the validity of the GLIM criteria in clinical applications.

Intervention strategies include comprehensive geriatric assessment, supportive interventions, nutritional counseling, food fortification, oral nutritional supplements (ONS), enteral nutrition (EN), parenteral nutrition (PN), and regular physical exercise for aged inpatients with malnutrition or at risk of malnutrition, according to the European Society for Parenteral and Enteral Nutrition consensus (14), American Society for Parenteral and Enteral Nutrition consensus (15), Integrated Caring for Older People (ICOPE) guidelines (16), and Chinese Society for Parenteral and Enteral Nutrition in geriatrics (17). Malnutrition management in the elderly is complicated and requires a multidisciplinary team. Johns Hopkins University established a multidisciplinary team to manage patients with malnutrition, which reduced the failure rate of nutritional screening by 47%, reduced inpatient expenses, and shortened hospital stays (18). Based on current literature, the establishment of a multidisciplinary and interdisciplinary nutrition support team (NST) and a multi-component malnutrition intervention strategy can ensure and improve the quality and safety of malnutrition treatment, ensure sufficient energy intake, and ameliorate nutritional condition and clinical outcomes (19, 20). Table 1 lists relevant studies (21–26).

**TABLE 1:** Studies on multi-component nutrition intervention or multidisciplinary team intervention for malnutrition in older adults.

First author	Patient type	*N*	Study intervention	Control treatment	Outcomes evaluated	Main effects of study intervention	Limitations	References
Ilana F	Hospitalized adults aged 65 and older at nutritional risk	259	Individualized nutritional treatment from a dietitian in the hospital and three home visits after discharge	Control group 1 received one meeting with a dietitian in the hospital. Control group 2 received standard care.	Mortality, health status, nutritional outcomes, blood tests, cognition, emotional, and functional parameters.	Lower mortality and moderate improvement in nutritional status were found in patients receiving individualized nutritional treatment during and after acute hospitalization.	Differences were observed in dropout rate across the study groups; high dropout rate.	(21)
Neelemaat F	Aged malnourished inpatients	210	A short-term oral nutritional intervention with protein and vitamin D and dietetic counseling	Usual care	Fat-free mass, hand grip strength, physical performance, and fall incidents	The number of patients who fall and fall incidents decreased	No blindness; the method of assessing participants’ nutritional intake was not optimal; high dropout rate	(22)
Neelemaat F	Aged malnourished inpatients	210	A short-term oral nutritional intervention with protein and vitamin D and dietetic counseling	Usual care	Quality adjusted life years, physical activities, and functional limitations	Significant improvement in functional limitations	The follow-up time was short	(23)
Beck AM	Older adults in nursing home and home-care		Multidisciplinary nutrition support (involving the physiotherapist, registered dietitian, and occupational therapist)	Usual care	Quality of life, physical performance, nutritional status, oral care, fall incidents, hospital admissions, rehabilitation stay, moving to nursing homes, and mortality.	A positive effect on quality of life, muscle strength, and oral care.	Nutrition assessment method was not selected Mini Nutritional Assessment; sample size was small.	(24)
Neelemaat F	Aged malnourished inpatients	210	A short-term oral nutritional intervention with protein and vitamin D and dietetic counseling	Usual care	1 and 4 year survival rates	The negative effects on long-term survival	No blindness	(25)
Schuetz P	Medical inpatients at nutritional risk	2,088	Individualized nutritional support	Standard hospital food	Any adverse clinical outcome	In medical inpatients at nutritional risk, the use of individualized nutritional support during the hospital stay improved important clinical outcomes, including survival, compared with standard hospital food.	No blindness; Protocol adherence issues; Nutrition might not be unconditionally generalizable to other health-care systems.in the control group; Did not yet investigate the costs of the intervention.	(26)

A recent systematic review of NST suggested that current studies lacked a robust evidence base because of the absence of well-designed research and good outcome measures (27). A meta-analysis on the efficacy of non-drug treatment in malnourished, aged patients indicated that there is a lack of high-quality evidence that shows which interventions are effective. There is an urgent requirement for high-quality research in this field (28).

## Methods and Analysis

### Study Aim

This study aims to assess the efficacy of a multidisciplinary team decision-making model on multiple interventions for aged, malnourished inpatients. We hypothesize that the multidisciplinary team intervention strategies may improve nutritional status, depression, functional status, and quality of life; reduce the incidence of frailty; and shorten hospital stays.

### Study Design and Setting

**FIGURE 1:**
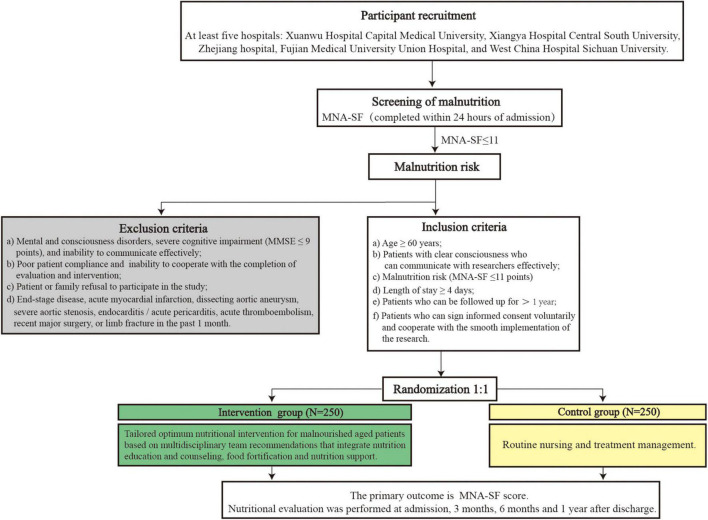
Flow diagram of the study. MNA-SF, Mini Nutritional Assessment Short Form.

This study is a prospective, multicenter, open-label randomized controlled trial. The multicenter trial includes at least five hospitals. The control group will undergo routine nursing and treatment management, in which the food served by the hospital cafeteria will depend on aged inpatient preferences, tastes, or abilities. The intervention group (hereafter also referred to as the case group) will be administered tailored optimum nutritional intervention for malnourished elderly patients based on multidisciplinary team recommendations that integrate nutrition education and counseling, food fortification, and nutritional support under NST guidance and supervision. Malnutrition risk screenings will be completed within 24 h of admission to the hospital. Follow-up investigations will be carried out at 3, 6 months and 1 year after discharge. The detailed flow diagram of the study is shown in Figure 1.

### Inclusion Criteria

Medical inpatients will be screened for malnutrition risk by physician staff using the MNA-SF within 24 h of admission (12, 29). Inpatients who meet the following criteria will be included: (a) Age ≥ 60 years; (b) patients with clear consciousness who can communicate with researchers effectively; (c) malnutrition risk (MNA-SF ≤ 11 points; (d) length of stay ≥ 4 days; (e) patients who can be followed up for > 1 year; and (f) patients who can sign informed consent voluntarily and cooperate with the smooth implementation of the research.

### Exclusion Criteria

Patients who meet the criteria listed below will be removed: (a) Mental and consciousness disorders, severe cognitive impairment (MMSE ≤ 9 points), and inability to communicate effectively; (b) poor patient compliance and inability to cooperate with the completion of evaluation and intervention; (c) patient or family refusal to participate in the study; and (d) end-stage disease, acute myocardial infarction, dissecting aortic aneurysm, severe aortic stenosis, endocarditis/acute pericarditis, acute thromboembolism, recent major surgery, or limb fracture in the past 1 month.

### Randomization

Eligible aged patients will be randomly 1:1 allocated into the control or intervention groups according to a pre-specified, computer-generated randomization scheme using SAS.

### Intervention Strategies

#### Responsibility and Interventions of Members of Nutrition Support Team

**FIGURE 2:**
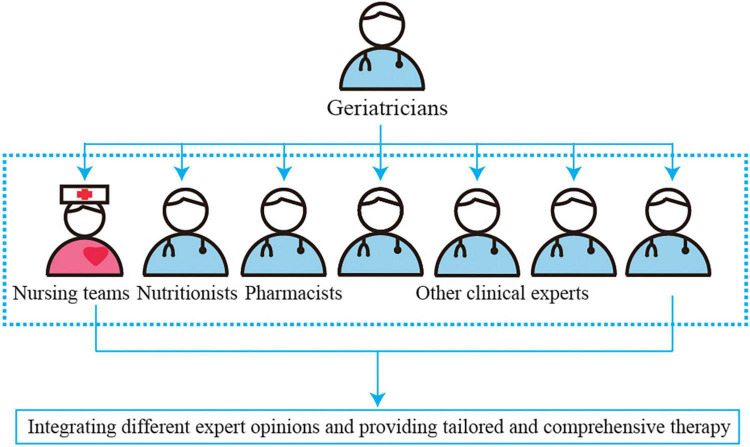
The clinical treatment model of nutrition support team (NST) to provide the tailored optimum nutritional intervention in malnourished aged patients based on multidisciplinary team recommendations.

The NST is composed of multidisciplinary experts in geriatrics, nutrition, nursing, pharmacy, rehabilitation, stomatology, surgery, neurology, cardiology, and others who provide technical support (19, 20) (Figure 2). Geriatricians play a major role in assisting, establishing, and managing the teams; integrating different expert opinions; and providing tailored and comprehensive therapy in the NST. The primary members of NST are nutritionists, clinical pharmacists, and nursing teams who implement the program. Table 2 shows the roles and interventions of the members of the NST.

**TABLE 2:** Responsibility and interventions of members of NST.

Member name	Roles and interventions
Geriatricians	● Play a major role in assisting, establishing, and managing the teams; integrating different expert opinions; and providing tailored and comprehensive therapy in the NST ● Assess the patient’s clinical and nutritional status ● Perform nutrition monitoring and reassess nutrition status of patients Review and document *multidisciplinary team recommendations* on nutritional or medical issues ● Formulate discharge nutrition prescription and assist family nutrition support ● Communicate with team members through consultation instructions and oral reports
Nursing teams	● Provide scientific nutrition education and related malnutrition science popularization for patients ● Provide medical care for patients, administer PN and EN and manage vascular passages ● Evaluate the changes of clinical and nutrition status of patients and monitor nutritional support complications ● Monitor the transition of care planning ● Communicate with team members through consultation instructions and oral reports
Nutritionists	● Provide the scientific nutrition education guidance for the inpatients ● Conduct comprehensive nutritional assessment of patients ● Calculate the appropriate nutrition targets and select optimum nutritional support methods ● Establish the nutritional therapy and care plan including feeding titration, feeding transition, and termination of nutritional support ● Monitor patients response to the nutrition therapy and EN and PN caused complications ● Adjust nutritional treatment plan based on individual conditions ● Communicate with team members through consultation instructions and oral reports
Clinical pharmacists	● Calculate the appropriate nutritional needs based on the comprehensive nutritional status of patients ● Develop the calculation, preparation, and administration plan of the intravenous nutrient ● Adjust nutritional treatment plan based on individual conditions ● Review and adjust medications related to nutrition problems ● Provide suggestions for supplementation of trace elements or special nutrients (i.e.., iron, vitamin B12, vitamin D, folic acid, calcium) based on individual conditions ● Communicate with team members through consultation instructions and oral reports
Other clinical experts	● Clinical experts in rehabilitation, stomatology, surgery, neurology, cardiology, and others who provide technical support, develop tailored nutritional interventions, and eliminate the causes of malnutrition as much as possible.

*NST, nutrition support team; EN, enteral nutrition; PN, parenteral nutrition.*

#### Nutritional Intervention Scheme

**FIGURE 3:**
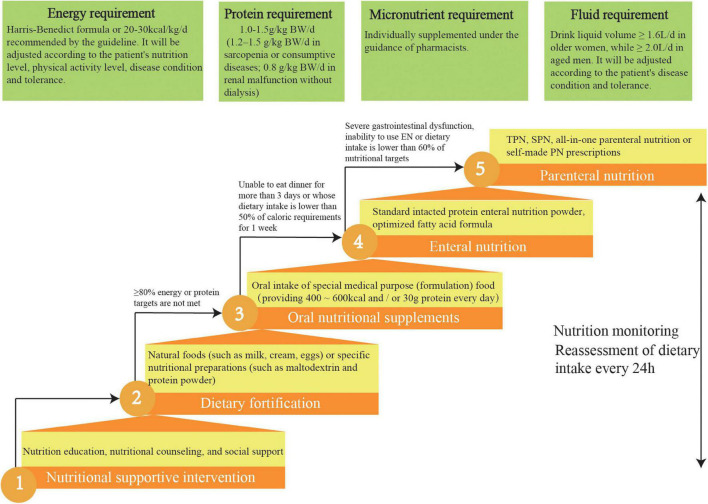
Five-steps-ladder nutritional approach used for the intervention group patients. TPN, total parenteral nutrition; SPN, supplementary parenteral nutrition.

We designed a practical nutritional intervention process by consensus and according to current guidelines (14–17) which clarified the nutritional strategies for the aged inpatients (Figure 3). We will apply the standard malnutrition clinical strategies for the elderly to the intervention patients. Different ladder treatments can be used simultaneously during the nutritional interventions.

#### Therapeutic Target of Nutritional Intervention

Intervention patients are required to meet four individual nutrition targets: ≥ 70% energy requirement, 90% fluid requirement, 100% protein requirement, and 100% micronutrient intake.

Daily energy requirements of patients are estimated before nutritional interventions. Currently, indirect calorimetry is recognized as the gold standard for assessing resting energy expenditure. Because of costly instruments and complicated operations, this study chose the most frequently used and accurate alternative method, the Harris–Benedict formula (30).


BMR⁢(womenEN)=655+[9.5×w⁢t⁢(k⁢g)]+[1.8×h⁢t⁢(c⁢m)]-(4.7×a⁢g⁢e)

BMR⁢(menEN)=66+[13.7×w⁢t⁢(k⁢g)]+[5×h⁢t⁢(c⁢m)]-(6.8×a⁢g⁢e)

where, wt is weight and ht is height.

According to current guidelines, the energy target for aged patients is 20–30 kcal/kg/d. Current literature indicates that aged, malnourished inpatients who meet this energy target can improve their prognoses and reduce mortality (14, 17). The energy target will be adjusted by the experts in the NST according to the patient’s nutrition level, physical activity level, disease condition, and tolerance. It is recommended that elderly patients with malnutrition consume larger amounts of high-quality protein (1–1.2 g/kg/d) (14, 17), with an even higher intake (1.2–1.5 g/kg/d) recommended for elderly patients with sarcopenia or consumptive diseases (31). However, patients with renal malfunction should reduce their protein intake.

In this study, micronutrients, such as serum ferritin, folic acid, vitamin B12, and serum 25 hydroxyvitamin D, for elderly patients at risk of and with malnutrition will be determined (4). According to these test results, they will be individually supplemented under the guidance of pharmacists. Clinical guidelines state that older women should drink ≥ 1.6 L, whereas older men should drink ≥ 2.0 L daily under the appropriate ambient temperature and general activity intensity, in the absence of special clinical conditions (such as heart failure or renal failure). Patients can choose drinks according to their preferences, such as water, tea, or coffee (14, 17).

##### Nutritional Education and Supportive Intervention

Nutritional education and supportive interventions for elderly patients are as follows: first, informing patients about the purpose of nutritional screenings and evaluations; second, answering questions from patients and their families about malnutrition; third, providing nutritional knowledge and a nutrient package for patients; and fourth, creating a soothing dining environment and encouraging patients to dine with others (14, 17). The pamphlets on nutrition jointly prepared by geriatricians and nutritionists will be delivered to the intervention group. On this basis, nutritionists will also carry out individualized nutrition education for patients in the intervention group. The intervention group will be provided nutritional counseling outside the hospital.

##### Dietary Fortification

A fortified diet refers to using natural foods (such as milk, cream, or eggs) or specific nutritional preparations (such as maltodextrin or protein powder) to strengthen the diet. Studies have shown that dietary fortification can boost energy and protein intake as well as improve clinical outcomes in the elderly. For aged patients, when food intake cannot meet 60% of the caloric requirement, ONS is the preferred nutritional intervention. The standard nutritional treatment for ONS is oral administration several times between meals, providing 400–600 kcal and/or 30 g protein/day (14, 17).

##### Enteral Nutrition

EN should be initiated immediately for patients who are expected to be unable to eat dinner for more than 3 days or whose dietary intake is lower than 50% of caloric requirements for 1 week. The standard intact protein powder is suitable for EN in most older inpatients. Long-term application of optimized fatty acid formulas can improve lipid metabolism and reduce cardiovascular events. Dietary fiber intake of ≥ 25 g/day is helpful in reducing constipation and diarrhea and in improving clinical outcomes (14, 17).

##### Parenteral Nutrition

Current guidelines recommend total PN for older patients with severe gastrointestinal dysfunction or the inability to use EN. For example, in aged inpatients where the energy and protein levels provided by EN are lower than 60% of the target energy requirement of the body, supplementary PN should be provided. All-in-one PN is recommended in elderly patients. This method can meet the physiological needs of the elderly, reduce the burden on the liver and kidney, reduce the concentration and osmotic pressure of a single nutrient, and reduce adverse events. For the elderly with special nutritional needs, self-made PN prescriptions can be issued to meet individual needs (14, 17).

##### Nutrition Monitoring

Nutrition monitoring will be performed in patients in the intervention group. The detection indicators include clinical symptoms and signs, energy supply compliance, laboratory examination (regular tests for electrolytes, blood glucose, blood lipid, plasma prealbumin, albumin, and C-reactive protein (CRP), according to the needs of the condition), and monitoring and management of EN and PN complications (14, 17).

### Control Group

The control group will undergo routine nursing and treatment management, in which the food served by the hospital cafeteria will depend on aged inpatient preferences, tastes, or abilities. The control group will receive pharmacological and non-pharmacological treatment according to the principles of “evidence-based medicine” and “clinical practice guidelines.” Nutritional treatment may be initiated in control patients at any time, but our nutritional support team will not take the initiative to provide multidisciplinary nutritional support.

### Outcomes

#### Primary Outcome

The primary efficacy outcome concerns the nutritional status of the participants. The MNA-SF score can be used to evaluate nutritional condition in individuals with various comorbidities (32). The MNA-SF is a screening tool with sensitivity and specificity in the highest quartile (>83 and >90%, respectively) for aged, malnourished inpatients (33). MNA-SF scores higher than 11 indicate that the nutritional status is considered normal and there is no risk of malnutrition. MNA-SF scores less than 11, however, indicate patients that are at risk of malnutrition (34). Investigators from the clinical staff will be trained to ensure the standardization and accuracy of data input, and follow-up assessments will be conducted at 3, 6, and 24 months after patient discharge through phone calls and at 12 months *via* face-to-face communication. The values during follow-up will be compared with those at baseline.

#### Secondary Outcomes

The following outcomes will be the secondary outcomes:

1. Anthropometric measurements [i.e., handgrip strength, calf circumference, weight, BMI, and fat-free mass index (FFMI)], blood biochemical analysis (i.e., routine blood tests, biochemical indexes, glycosylated hemoglobin, serum 25- hydroxyvitamin D concentration, folic acid, vitamin B12, ferritin, CRP), hospital costs and stay, hospital readmissions and all-cause mortality rate will be assessed.

2. Functional status using the Activities of Daily Living (ADL) scale (35) and functional impairment screening tool (FIST) (36); cognition using the Mini Mental State Examination (MMSE) score (37); depression *via* the 15-item Geriatric Depression Scale (GDS-15) (38); quality of life using the 12-item Short Form Survey (SF-12) score (39); physical function status using the short physical performance battery protocol (SPPB) (40); frailty *via* frailty phenotype (41) and frailty screening questionaire (FSQ) (42); social frailty (SF) *via* HALFT scale (43) and intrinsic capacity (IC) *via* WHO ICOPE screening tool (44) will be evaluated, as presented in the following section.

### Participant Schedule and Assessment Tools

A structured comprehensive assessment of patient functional status, cognition, mood, physical performance, quality of life, and the condition of frailty will be performed twice during the course of the study. The first baseline assessment is to be conducted on the day of group allocation, and a reassessment will be performed 12 months after discharge. Current guidelines recommend that the nutritional condition of aged inpatients be comprehensively evaluated based on clinical severity stratification, food intake, weight and body composition, and comprehensive geriatric assessment.

The MNA-SF is a highly sensitive and specific screening tool for malnutrition in the elderly. It determines the nutritional status of patients according to six questions regarding medical history, weight, eating status, and simple physical examination, and has a total possible score of 14 points. The scoring criteria are as follows: malnutrition (0–7 points), at risk of malnutrition (8–11 points), and normal nutritional status (12–14 points) (45).

The association of at least one phenotypic criterion and one etiologic criterion can suffice the diagnostic requirements of malnutrition based on the GLIM criteria (12). Table 3 shows the diagnostic criteria for malnutrition based on the GLIM criteria. The phenotypic criteria include non-volitional weight loss, low BMI, and reduced muscle mass. Fat-free mass by bioelectrical impedance analysis (BIA) is measured using a body composition analyzer (Hongshentai, IOI353, China). The fat-free mass is divided by the squared height (kg/m^2^) to obtain the FFMI.

**TABLE 3:** Diagnostic criteria for malnutrition based on the GLIM criteria.

Phenotypic criterion					Etiologic criterion	
Non-volitional weight loss	Low BMI (kg/m^2^)	Reduced muscle mass			Reduced food intake or assimilation	Disease burden/inflammation
>5% within past 6 months, or >10% beyond 6 months	<18.5 if <70 years, or <20 if >70 years		**M**	**F**	The scores of “0” or “1” on the first MNA-SF item will be considered as positive, or ask about any GI symptoms/condition that adversely impacts food assimilation or absorption through the questionnaire.	Acute disease or injury, or chronic disease-related or CRP > 10 mg/L
				
		FFMI (kg/m^2^)	< 17	<15		
		**AWGS 2019**				
		HGS(kg)	<28	<18		
		CC (cm)	<34	<33		

*M, Male; F, Female; BMI, body mass index; FFMI, Fat free mass index; AWGS, Asian Working Group for Sarcopenia; HGS, hand grip strength; CC, calf circumference; CRP, C-reactive protein.*

Considering the equipment conditions in different clinical research centers and the safety of patients with respect to cardiac implantation equipment (such as cardiac pacemakers), if BIA is not available, physical testing or standard anthropometric measurements (e.g., calf circumference) and functional assessments (e.g., handgrip strength) recommended by the GLIM may be considered supportive measures (11). The thresholds values for calf circumference and handgrip strength recommended by Asian Working Group for Sarcopenia (46) are used: calf circumference of < 34 cm and handgrip strength of < 28 kg in men, calf circumference of < 33 cm and handgrip strength of < 18 kg in women. Handgrip strength, expressed in kilograms, will be measured using a handheld dynamometer (CAMRY, EH101, China).

The etiologic criteria include reduced food intake or assimilation, disease burden, and inflammation. The first MNA-SF item is employed to determine reduced food intake, and asks if food intake has declined over the past 3 months due to loss of appetite, digestive problems, chewing, or swallowing difficulties, with scores of “0” or “1” considered as positive (47). Gastrointestinal symptoms (such as dysphagia, nausea, vomiting, diarrhea, constipation, or abdominal pain) or chronic conditions (such as short bowel syndrome, pancreatic insufficiency, post-bariatric surgery, esophageal strictures, gastroparesis, or intestinal pseudo-obstruction) that detrimentally impact food assimilation or absorption will also be considered. Disease burden includes acute disease or injury-related conditions (including major infection, burns, trauma, or closed head injuries) and chronic disease-related conditions (including malignant disease, chronic obstructive pulmonary disease, congestive heart failure, chronic renal disease, or any disease with chronic or recurrent inflammation). CRP is selected as a biomarker to assess inflammation, as recommended by the GLIM. CRP levels of > 10 mg/L are considered positive (48).

Functional performance will be assessed using the ADL scale (35), which comprises domains such as independence in feeding, bathing, dressing, functional mobility, bladder control, and bowel movements. Using the ADL scale, functional impairment will be assessed as follows: mild (61–99); moderate (41–60); and severe (< 40). FIST will be used to evaluate physical function. It includes three domains: ability to perform activities of daily living, ability to engage in domestic life, and ability to engage in social activities (36). The FIST total score is 0–16. The higher the points are, the better the physical function is.

The MMSE was invented by Folstein et al. (37) and is now applied widely and successfully in cognitive screenings for the elderly. It primarily evaluates orientation, registration and recall, language ability, calculation, and attention. The patients are scored between 0 and 30 points. MMSE scores of greater than 27 indicate normal cognitive function, while scores less than 27 indicate cognitive impairment. According to the scoring standard, the patient may also be classified as having severe dementia (≤10 points), moderate dementia (10–20 points), or mild dementia (≥21 points).

The GDS-15 scale will be used to evaluate depressive status in the last week, with scores ranging from 0 to 15 (38). GDS-15 scores greater than 5 indicate that the patient may be prone to depression.

The SF-12 is a simplified alternative version of the SF-36 health survey, originally developed in the United States (39). The higher the SF-12 score, the better the quality of life.

The WHO recommends using the SPPB test to assess functional capacity in the elderly (40). There are three parts to the test: balance assessment, 4-m walking assessment, and the get-up and sit test. The three parts together add up to the final score. According to the score standard, patients may be regarded as having serious limitation (0–4 points), moderate limitation (5–6 points), slight limitation (7–9 points), and minimal limitation (10–12 points).

The frailty phenotype assessment was developed by Fried et al. (41) and comprises the following criteria: weakness, slow gait speed, low physical activity, exhaustion, and unintentional weight loss. According to the assessment, aged patients may be identified as frail (≥3 criteria present), pre-frail (1–2 criteria present), or robust (0 criteria present). The FSQ scale was developed by Ma et al. (42). It consists of 4 components: slowness, weakness, inactivity, and exhaustion, with scores ranging from 0 to 4. A score of 0 may be indicated robust; 1–2 may be indicated pre-frail; and a score of ≥ 3 may be considered frailty. SF will be assessed by HALFT scale (43). The HALFT scale includes the following 5 items: inability to help others, limited social participation, loneliness, financial difficultly, and not having anyone to talk to, with scores ranging from 0 to 5. According to the score standard, patients may be regarded as non-SF (0 score), pre-SF (1–2 scores), and SF (≥3 scores).

IC will be evaluated by WHO ICOPE screening tool (44) and consists of the following five items: cognitive decline, limited mobility, malnutrition, sensory loss, and depressive symptoms, with scores ranging from 0 to 6. Higher score indicates better IC.

**FIGURE 4:**
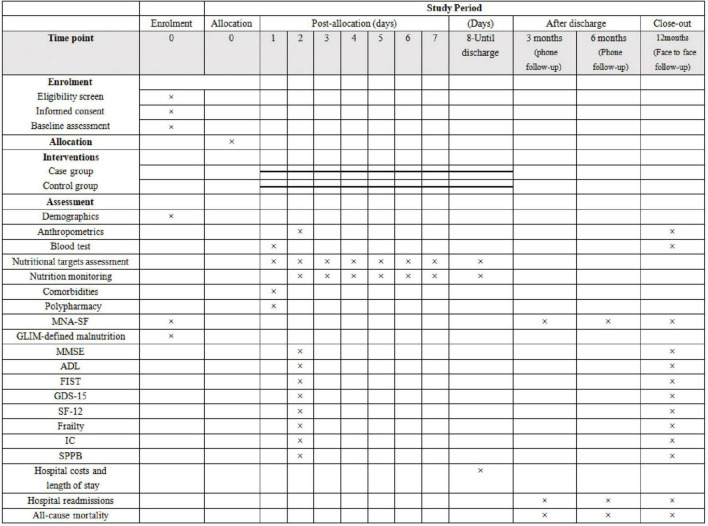
Diagram for time schedule of enrolment, allocation, and assessment. MNA-SF, Mini Nutritional Assessment Short Form; GLIM, the Global Leadership Initiative on Malnutrition; MMSE, Mini-Mental State Examination; ADL, Activities of Daily Living scale; FIST, functional impairment screening tool; GDS-15, the 15-item geriatric depression scale; SF-12, the 12-item short form health survey; IC, intrinsic capacity; SPPB, short physical performance battery.

A diagram of the schedule of aged patient enrolment, allocation, and assessment is shown in Figure 4. In this study, sociodemographic and clinical data of the patients as well as information on various comorbidities and polypharmacy will be collected during patient hospitalization.

### Sample Size Calculation

The 3M study is a parallel, randomized controlled study. It will adopt a ratio of 1:1 between the case and control groups. The MNA-SF score is the primary outcome index. According to the literature, the mean MNA-SF value in the control group was estimated to be 12.1 ± 1.9 (49). After the intervention, the expected average difference in MNA-SF scores between the two groups will be 1.5 (50). The sample size was estimated using a bilateral test. The calculation is based on an alpha level of 0.05 and a desired power of 90%. The sample size of N1 and N2 was 34 cases in the case and control groups, and was calculated using the PASS (version 11.0.7; PASS, NCSS, LLC., Kaysville, Utah, United States). Assuming that the dropout rate is 20%, the sample size is as follows: *N*1 = *N*2 = 34 ÷ 0.8 = 43. Considering the long follow-up time, we will enroll at least 50 participants in each group. A total of 500 patients will be included.

### Data Gathering, Confirmation, and Management

Clinical trial supervisors will periodically scrutinize the extracted data for validity and accuracy. All clinical data collection and comprehensive assessments will be performed by uniformly trained investigators. The investigators will faithfully fill clinical report forms according to study protocol requirements. All participant information will be kept strictly confidential and not divulged to any individual or organization.

### Statistical Analysis

Epidate 3.1 will be used for data entry, and Excel will be used to establish the database. All data will be checked, numbered, and entered by two researchers. The primary outcome, that is, the MNA-SF score, will be compared between the intervention and control groups using analysis of repeated measurements of variance. The Student’s *t*-test or Mann–Whitney *U*-test will be performed to evaluate the statistical differences between the intervention and control groups in the matter of functional status, cognition, quality of life, mood level, physical functional capacity, and length of stay, and the normality of the distributions will be tested using the Shapiro–Wilk test. The incidence of malnutrition and frailty will be compared between the intervention and control groups using chi-square statistics. Survival analyses will be conducted using intention-to-treat analysis by log-rank tests and Cox proportional-hazards model adjusted for confounders. *P*-value of < 0.05 will be considered for statistical significance. All statistical analyses will be performed using SPSS.

### Ethics Approval and Consent to Participate

This study was approved by the ethics review board of Xuanwu Hospital of Capital Medical University (2021-060) and all the patients will sign written informed consent.

## Discussion

As the population ages, society faces enormous challenges related to advancing age, deteriorating health, various comorbidities, and polypharmacy, which can lead to disability, dependence, and economic burden (51, 52). One significant challenge is malnutrition. Malnutrition is common but easily ignored in older, hospitalized patients. Malnutrition is reversible, but older adults may experience adverse consequences if it is not diagnosed and treated at an early stage. Early detection of individual nutritional risks and timely initiation of appropriately tailored nutritional therapy are essential in the elderly. The etiology of malnutrition is often multifactorial in aged patients (53), and multi-professional nutritional support teams are required to ensure and improve the quality and safety of nutritional therapy.

The first report of NST in organizing nutritional support was published in 1972, in response to the high incidence of sepsis in hospitalized patients with PN (54). According to current literature, large-scale, multicenter, high-quality clinical trials are insufficient in various fields of clinical nutrition, including NSTs. Most of the current evidence is based on observational studies and the clinical experience of doctors and nutritionists. Recently, some new and important clinical trials have provided strong evidence in favor of NST (26, 55).

Thorne and Baldwin attempted to verify the theory that using multidisciplinary interventions may improve the nutritional status, quality of life, functional status, and survival in malnourished inpatients or in those at risk of malnutrition, through a meta-analysis (56). However, the research involved a broad group of patients and could not therefore be proven as valid for aged inpatients specifically. A recent systematic review showed that multidisciplinary nutritional support was associated with lower mortality and higher quality of life in older patients (57). However, the number of studies included was relatively small. Thus, a more high-quality randomized control study is required to verify these findings and provide a more reliable basis for clinical practice.

In this 3M study, the GLIM criteria will be used for the diagnosis of malnutrition in the elderly and to guide clinical nutritional intervention to compensate for the lack of interventional research. Guided by the concept of interdisciplinary cooperation, the 3M study will establish a multidisciplinary NST; develop an innovative intervention strategy, integrating nutritional education and consultation, dietary fortification, and nutrition support and monitoring; carry out nutrition screenings, comprehensive nutrition evaluations, and nutrition therapy for hospitalized aged inpatients with malnutrition or those at risk of malnutrition; and formulate the clinical pathway for malnutrition, standardized diagnosis, and treatment of malnutrition in older inpatients. If this project is successful, we hope to promote clinical pathways in hospitals nationwide in order to improve the nutritional status of aged patients and reduce poor prognoses.

There are some limitations to the study. First, due to the nutritional intervention strategies of this study, including nutrition education and consultation, it is impossible to blind the participants and investigators. Second, the study strategy is based on the use of individualized comprehensive therapies based on the NST. It may not be possible to analyze the impact of each intervention on the clinical outcome of elderly patients, but we will record the nutritional therapy of each patient in the case group. Finally, patients in the intervention group may have poor compliance. The NST provides individual nutritional therapy for patients, but not all patients can reach the therapeutic target (including energy, fluid, protein, and micronutrient targets). We will record the actual nutritional intake in the form of a nutrition diary.

Currently, there is shortage of clinical standard pathways and management modes for malnourished elderly patients in China. We propose a comprehensive and pragmatic study protocol that aims to assess the effectiveness of tailored optimum nutritional intervention in malnourished elderly patients. These nutritional interventions will be based on multidisciplinary team recommendations that may further develop standardized strategies. We hope that the implementation of this protocol would improve the nutritional, functional, and mood status, as well as reduce the incidence of frailty, enhance quality of life, shorten hospital stays, and improve mortality in elderly patients.

## Data Availability Statement

The original contributions presented in the study are included in the article/supplementary material, further inquiries can be directed to the corresponding author/s.

## Ethics Statement

This study was approved by the ethics review board of Xuanwu Hospital of Capital Medical University (2021-060). The patients/participants provided their written informed consent to participate in this study.

## Author Contributions

TJ wrote the manuscript. LM and YL designed the study, revised the manuscript, and supervised the work. TJ, LZ, RH, LP, SS, XL, YS, XC, QC, and 3M Study Working Group collected and collated data. All authors contributed to the article and approved the submitted version.

## Conflict of Interest

The authors declare that the research was conducted in the absence of any commercial or financial relationships that could be construed as a potential conflict of interest.

## Publisher’s Note

All claims expressed in this article are solely those of the authors and do not necessarily represent those of their affiliated organizations, or those of the publisher, the editors and the reviewers. Any product that may be evaluated in this article, or claim that may be made by its manufacturer, is not guaranteed or endorsed by the publisher.
